# Thirty years of the Family Health Strategy in the Brazilian Unified National Health System: milestones, advances, setbacks and challenges

**DOI:** 10.1590/0102-311XEN206025

**Published:** 2026-06-22

**Authors:** Ligia Giovanella, Maria Helena Magalhães de Mendonça, Estela Márcia Saraiva Campos, Ana Luiza Queiroz Vilasbôas, Rosana Aquino, Luiz Augusto Facchini

**Affiliations:** 1 Centro de Estudos Estratégicos da Fiocruz Antonio Ivo de Carvalho, Fundação Oswaldo Cruz, Rio de Janeiro, Brasil.; 2 Escola Nacional de Saúde Pública Sergio Arouca, Fundação Oswaldo Cruz, Rio de Janeiro, Brasil.; 3 Faculdade de Medicina, Universidade Federal de Juiz de Fora, Juiz de Fora, Brasil.; 4 Instituto de Saúde Coletiva, Universidade Federal da Bahia, Salvador, Brasil.; 5 Departamento de Medicina Social, Universidade Federal de Pelotas, Pelotas, Brasil.

**Keywords:** Family Health Strategy, Primary Health Care, Unified Health System, Estrategia de Salud Familiar, Atención Primaria de Salud, Sistema Único de Salud

## Abstract

This essay examines the trajectory and persistent challenges faced by Brazil’s Family Health Strategy (FHS), stemming from concerns regarding the prospects for the full implementation this territory-based community-oriented approach to the delivery of primary health care within the Brazilian Unified National Health System. We look at the development of the strategy and provide a critical overview of the main institutional milestones over the last 30 years, focusing on the following: federal funding associated with the process of the municipalization of care; the expansion of multidisciplinarity in the work of health professionals in basic health units (BHU) and the local community; improvements in quality associated with the evaluation of team structure and work processes and performance-based funding; and emergency medical staffing in underserved areas and professional training. We outline how the expansion of FHS coverage and the adoption of practices centered on individuals, families and communities has impacted population health and access to care. Setbacks between 2016 and 2022 are critically addressed, including the undermining of the multidisciplinary community-based approach and threats to universality and comprehensiveness. We then go on to discuss the reprioritization of the FHS by the Federal Government in 2023. Data from the 2024 national BHU Census are used to illustrate the current status of the FHS. Challenges to implementing effective change in the care model and achieving universal FHS coverage include the following: ensuring adequate funding, employment stability, professional training and interprofessional collaboration; addressing the risks of commodification in primary health care (PHC); improving coordination of care and the strengthening the role of PHC in patient access management; and reducing geographical, social and racial inequalities in access to care.

## Abstract (es)

Este ensayo trata de la trayectoria y de los retos persistentes de la Estrategia de Salud Familiar (ESF) en Brasil, a partir de inquietudes sobre las perspectivas para la plena implementación de este enfoque de atención primaria de salud de base territorial y orientación comunitaria en el Sistema Único de Salud brasileño (SUS). Examina la trayectoria de expansión y revisa críticamente los principales hitos institucionales de la ESF en estos 30 años, destacando: la inducción financiera federal asociada al proceso de municipalización; la ampliación de la multiprofesionalidad en el trabajo desarrollado en las unidades básicas de salud (UBS) y territorios; la inducción de la calidad articulada a la evaluación de la estructura y el proceso de los equipos y transferencias financieras asociadas al rendimiento; y la provisión de emergencia de médicos en áreas desatendidas asociada a la formación profesional. Sintetiza las repercusiones de la expansión de la cobertura de la ESF y la adopción de prácticas centradas en las personas, las familias y las comunidades sobre la salud y el acceso. Se abordan críticamente los retrocesos entre 2016 y 2022, con restricciones al enfoque comunitario multiprofesional y amenazas a la universalidad y la integralidad, así como la reanudación, por parte del Gobierno Federal, de la prioridad para la ESF a partir de 2023. Los datos del Censo de las UBS 2024 ilustran la situación actual resultante de esta trayectoria. En el análisis de los retos para el cambio efectivo en el modelo asistencial y la universalización de la ESF, destacan la suficiencia de la financiación;la estabilidad funcional, la formación y el trabajo colaborativo interprofesional;los riesgos de mercantilización en la atención primaria de salud (APS); la coordinación de la atención y el papel de la APS en la regulación asistencial;y las desigualdades geográficas, sociales y étnico-raciales en el acceso.

## Introduction

Referred to as primary care in national policy as opposed to selective and focused care ^1^, primary health care (PHC) in Brazilian Unified National Health System (SUS, acronym in Portuguese), has witnessed a qualitative leap forward, starting in 1994 with the gradual implementation of the Family Health Program, now called the Family Health Strategy (FHS).

This process marked an inflection point for Brazil’s healthcare model, with the adoption of a community-oriented, territorialized approach integrated into the health network and implemented by multidisciplinary health teams. A large body of evidence shows that the FHS has achieved broad population coverage, improved access and health indicators and reduced inequalities ^2^ ^,^ ^3^ ^,^ ^4^.

Despite these advances, the FHS has yet to realize its full potential, being constrained by financial and infrastructure barriers and high rates of staff turnover. Between 2016 and 2022, historical weaknesses were intensified by the political and institutional landscape, undermining the universality, equity and comprehensiveness of the SUS ^5^.

In the wake of the celebration of the 30th anniversary of the FHS, this essay stems from concerns regarding the future and prospects for this globally recognized innovative PHC model ^6^, and addresses the main challenges in achieving its full implementation. To this end, we present a critical analysis and revisit the main historical and institutional milestones achieved during the expansion of the FHS since 1994, with the incorporation of oral health care and the creation of the Family Health Support Centers (NASF, acronym in Portuguese; subsequently Family Health Expanded Centers), Brazilian National Program to Improve Access and Quality in Primary Care (PMAQ-AB, acronym in Portuguese) and More Doctors Program (PMM, acronym in Portuguese). We then go on to discuss how the expansion of FHS coverage and adoption of practices centered on the individual, family and community has impacted access to healthcare and population health. Setbacks during the period 2016-2022 are then critically examined, together with the reprioritization of the FHS by the Federal Government in 2023. An overview of the current status of PHC in Brazil is complemented with recent data from the 2024 Brazilian National Basic Health Unit Census (BHU Census), which also illustrate the challenges highlighted above.

Finally, we examine the persistent challenges in consolidating the FHS model, including adequate funding to ensure universal coverage, the commodification in PHC, strengthening training and continuing education, stable employment relationships and careers in health, care coordination and continuity, integration of PHC into care networks, and the reduction of geographical, social and racial and ethnic inequalities.

This essay seeks to contribute to the debate on the future of the FHS, reinforcing the need to strengthen the strategy as a key mechanism for upholding the principles of the SUS in the face of contemporary demographic, epidemiological and social changes.

## The trajectory of primary health care in the SUS

With the creation of the SUS in 1988, guided by the underlying principle of decentralization, municipalities gradually assumed responsibility for PHC through the provision of basic care services and changes in the care model brought about by federal regulations and incentives and local initiatives. As part of the expansion process, an extensive network of basic health units (BHU) was established across the country. In 2024, this network comprised approximately 45,000 BHU distributed across the country’s 5,570 municipalities, ensuring universal, free access to primary care.

The following describes and analyzes the main historical and institutional milestones in this trajectory.

### The transformation of the Family Health Program into a strategy

Created in 1994, the Family Health Program introduced a community- and territory-based approach, integrating PHC into the SUS network through multidisciplinary teams that provided continuous care to individuals, families and communities, including strategies to strengthen patient affiliation and emphasizing the central role of community health workers (CHW).

Originally, teams consisted of a general practitioner, nurse, nursing technician or assistant and four to six CHW, selected from the local community. CHW play a key role in developing activities in the local community, performing routine home visits to around 150 families living in micro-areas. They register families, carry out community appraisals and implement health protection measures, identifying at-risk groups ^7^.

Health teams are therefore also responsible for disease prevention and surveillance and health promotion, with an emphasis on the social determinants of health and illness, which includes intersectoral actions and public participation as part of interventions tailored to the specific characteristics of each territory and according to the intrinsic characteristics of managers, professionals and patients ^8^.

With the publication of *Basic Operating Standard n. 01/1996*, the Family Health Program began to play a pivotal role in the organization of PHC, receiving specific federal funding and with the aim of upholding the principles of the universality and equity of access to health services for all the population. The municipal government became responsible for the implementation and management of PHC, bringing health care services as close as possible to where people live ^9^.

Federal transfers played a decisive role in promoting the FHS and in the decentralization of the SUS. The Fixed Primary Health Care Floor (PAB, acronym in Portuguese) ensured municipal government autonomy, while the variable PAB facilitated the expansion of FHS teams, consolidating the community-oriented model, which was integrated into the health care network, positively affecting population health. In 2006, the Brazilian National Primary Health Care Policy (PNAB, acronym in Portuguese) renamed the Family Health Program as the FHS and confirmed the strategy’s pivotal role as a substitute for the traditional primary care model ^10^ ^,^ ^11^ ^,^ ^12^.

Initially selective and focused on expanding PHC coverage in underserved areas, the Family Health Program gradually reached large urban centers. In large cities, especially in the country’s Central-West, Southeast and South regions, the strategy consisted of converting traditional BHU to the new FHS model, without necessarily increasing population coverage ^2^ ^,^ ^7^ ^,^ ^13^ ^,^ ^14^. Between 2003 and 2009, this process was supported by the Family Health Expansion and Consolidation Project (PROESF, acronym in Portuguese). The baseline studies carried out under this project played a key role in promoting the institutionalization of PHC monitoring and evaluation, in tandem with the creation of the Primary Health Care Monitoring and Evaluation Coordination Unit within the Primary Health Care Department of the Health Care Secretariat of the Brazilian Ministry of Health ^14^.

The 2006, 2011 and 2017 versions of the PNAB introduced changes to the scope of health teams and coverage parameters. The 2011 PNAB expanded the scope of family health to include specific modalities for vulnerable populations served by riverine health teams, floating BHU and mobile clinics, adopted PHC as a synonym for primary care and reduced the recommended team catchment population from 4,000 to an average of 3,000, or less for more vulnerable populations. The 2017 PNAB increased the catchment population to an average of 3,500 people and unfortunately discontinued the exclusive financial incentive for the FHS ^5^.

**Figure 1:**
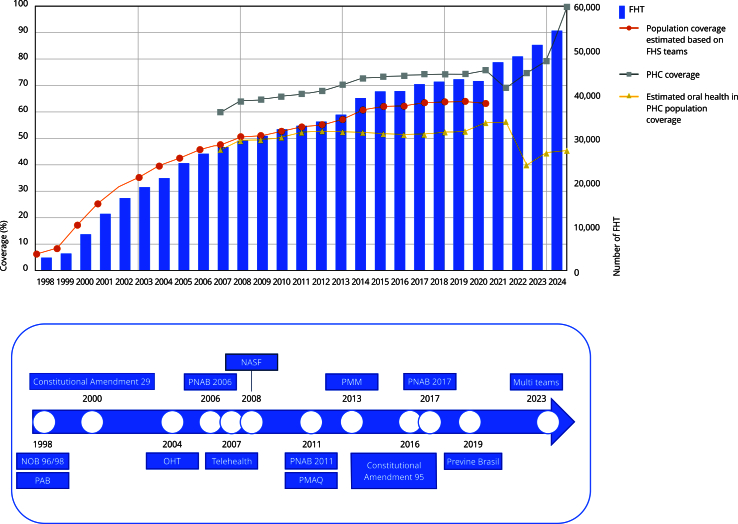
Timeline showing key milestones in Brazilian National Primary Health Care Policy (PNAB, acronym in Portuguese) and expansion of family health teams (FHT) and estimated primary health care team (PHCT) and oral health teams (OHT) in primary health care (PHC) population coverage, according to the Brazilian Ministry of Health’s Primary Health Care Department. Brazil, 1998-2024.

Nevertheless, the FHS has established itself as a key pillar of PHC in Brazil. However, uneven implementation and persistent structural challenges highlight the need for more investment in training, stable funding and integration of PHC into the health care network to maximize effectiveness ^7^. The evolution of coverage and the main milestones in the history of the FHS are presented in Figure 1 and Table 1.

NASF: Family Health Expanded Centers; NOB: *Basic Operating Standard*; PMAQ-AB: Brazilian National Program to Improve Access and Quality in Primary Care; PMM: More Doctors Program. Source: Brazilian Ministry of Health e-Gestor (https://egestoraps.saude.gov.br/) primary care information system, considering public reports including time series data on number of funded teams and services, PHC coverage, oral health coverage, PHC funding and community health worker (CHW) coverage, based on the availability of indicators and methodology used for each period (1998-2024). Note: the “FHS team population coverage” indicator was discontinued in 2020. In 2021, the “PHC coverage” indicator began to be used, calculated based on the estimated population registered by FHT and PHCT funded by the Brazilian Ministry of Health. In 2024, the “Potential PHC coverage” indicator was adopted, which includes FHT, PHCT, street clinic teams (SCT), family health teams for riverine communities (RFHT) and prison primary health care teams (PPHCT) co-funded by the Ministry of Health and teams funded by state and municipal governments. In 2024, a new method was adopted for the “estimated oral health in primary health care population coverage” indicator, based on the oral health strategy population coverage linked exclusively to the FHS, including SCT, RFHT and PPHCT.

**Table 1:** Evolution of population coverage * and number of Family Health Strategy (FHS) teams, oral health teams (OHT), Family Health Expanded Centers (NASF) and community health workers (CHW). Brazil, 1998-2024.

Year	FHT (n)	PHCT (n)	Estimated population coverage by the FHS teams (%)	PHC population coverage (%)	OHT (n)	Estimated population coverage by OHT linked to FHT (%)	Estimated population coverage by oral health in PHC (%)	NASF (n)	CHW (n)
1998	3,062	-	6.6	-	0	-	-	0	-
1999	4,114	-	8.8	-	0	-	-	0	-
2000	8,503	-	17.6	-	0	-	-	0	-
2001	13,155	-	25.6	-	2,248	-	-	0	-
2002	16,698	-	32.1	-	4,261	-	-	0	-
2003	19,068	-	35.7	-	6,170	-	-	0	-
2004	21,232	-	39.9	-	8,951	-	-	0	-
2005	24,564	-	42.8	-	12,603	-	-	0	-
2006	26,729	-	46.2	-	15,086	-	-	0	-
2007	28,195	-	48.0	60.3	17,508	29.9	46.2	0	245,663
2008	29,769	-	51.0	64.3	19,280	33.3	49.5	0	257,578
2009	30,898	-	51.4	65.0	20,626	34.6	49.8	0	261,839
2010	32,243	-	53.0	66.2	21,999	36.5	50.8	1,317	270,775
2011	33,135	-	54.6	67.1	23,076	38.4	52.7	1,564	275,246
2012	33,899	-	55.5	68.3	23,586	38.9	53.0	1,929	283,404
2013	35,567	-	57.5	70.6	24,131	39.4	52.9	2,767	284,136
2014	39,426	-	61.1	73.1	25,327	39.8	52.5	3,898	288,335
2015	40,765	-	62.5	73.7	25,891	40.3	52.1	4,288	285,736
2016	41,061	-	62.6	74.1	25,827	39.9	51.7	4,406	286,556
2017	42,513	-	63.9	74.6	27,082	41.2	51.9	4,886	284,867
2018	43,016	-	64.2	74.6	28,050	42.1	52.7	5,517	285,570
2019	43,755	-	64.5	74.8	28,991	43.1	53.0	5,487	284,196
2020	43,286	-	63.6	76.1	30,606	45.0	56.1	-	287,064
2021	47,501	3,869	-	69.5	29,816	46.1	56.6	-	288,771
2022	48,817	3,712	-	75.1	28,054	-	40.4	-	291,378
2023	51,369	5,708	-	79.7	31,035	-	44.8	-	295,406
2024	54,629	6,355	-	100.0	34,591	-	45.9	4,567 **	300,104

FHT: family health teams; PHC: primary health care.

Source: Brazilian Ministry of Health e-Gestor (https://egestoraps.saude.gov.br/) primary care information system, considering public reports including time series data on number of funded teams and services, PHC coverage, oral health coverage, PHC funding and CHW coverage, based on the availability of indicators and methodology used for each period (1998-2024).

* The “FHS team population coverage” indicator was discontinued in 2020. In 2021, the “PHC coverage” indicator began to be used, calculated based on the estimated population registered by FHT and PHCT funded by the Brazilian Ministry of Health. In 2024, the “Potential PHC coverage” indicator was adopted, which includes all types of teams co-funded by the Brazilian Ministry of Health, as well as teams funded by state and municipal governments. In 2024, a new method was adopted for the “Estimated oral health in PHC population coverage” indicator, based on the oral health strategy population coverage linked exclusively to the FHS, including street clinic teams (SCT), family health teams for riverine communities (RFHT) and prison primary health care teams (PPHCT);

** Includes resumption of federal funding for multidisciplinary teams in PHC under the multidisciplinary teams model.

### Smiling Brazil Program and oral health teams

Starting in 2000, two types of oral health teams (OHT) were gradually incorporated into the FHS: one consisting of a dentist and dental assistant; and the other consisting of a dentist and dental assistant plus a dental technician. In 2004, during the first Luiz Inácio Lula da Silva administration, the government launched the Smiling Brazil Program, creating the basis for the Brazilian National Oral Health Program (PNSB, acronym in Portuguese). The program aimed to improve access to services and increase the comprehensiveness of care through individual and collective oral health promotion, prevention, diagnosis, treatment and rehabilitation actions, including the expansion of water fluoridation and oral health surveillance in the SUS ^15^.

The PNSB also structured a network of specialized services through the creation of specialized dental centers (CEO, acronym in Portuguese), regional dental prosthesis laboratories (LRPD, acronym in Portuguese), and, more recently, specialized oral health services (SESB, acronym in Portuguese), aimed at small municipalities.

Federal policy guidance and financial incentives brought about significant changes in the healthcare model and led to greater integration of dental care into PHC. The number of oral health teams co-financed by the Brazilian Ministry of Health increased from 8,900 in 2004 (Table 1) to 19,280 in 2008, in tandem with a significant fall in the number of people in the country who had never been to a dentist ^15^.

Despite a significant reduction in barriers to access, OHT coverage remained lower than that of FHT, with coverage stagnating between 2019 and 2022 (Table 1) and actions experiencing a number of setbacks ^16^ (Table 1 and Figure 1).

The institutionalization of the PNSB by *Law n. 14,572/2023* ^17^ represented a milestone for the sustainability of national oral health policy, with the SUS standing out from other countries with universal health systems, which generally do not provide oral health services. Nevertheless, challenges remain, including strengthening interprofessional work and balancing attention to individual demands and collective health promotion.

To this end, it is crucial to invest in technological innovations, such as integrated electronic health records, teledentistry and digital epidemiological monitoring tools, enhancing team capacity to address health needs and deliver effective solutions and coordination of care with the services provided by specialized care network. It is also necessary to increase OHT coverage in line with FHS coverage, reducing regional and social inequalities ^17^.

### Expansion of multidisciplinarity: Family Health Expanded Centers

Launched in 2008 through federal incentives, NASF were designed to expand multidisciplinarity within the FHS by including health professionals from different areas and specialties, such as psychologists, nutritionists, physical therapists, social workers, physical educators, psychiatrists, and strengthening matrix support for primary care teams. Their purpose was to broaden the scope of actions, enhance the capacity of BHU to address health needs and deliver effective solutions and promote interprofessional practices through shared care, continuing education and interventions in the local community ^18^. However, implementation was uneven, often reproducing outpatient models centered on individual care or managerial functions to the detriment of integrated collaboration ^18^.

In 2019, NASF were defunded by the Brazilian Ministry of Health amid an increasing emphasis on the centrality of medical work, compromising multidisciplinarity in PHC ^5^. In 2023, the NASF program was reformulated on the back of the rebuilding of the SUS and a renewed emphasis on the priority of the FHS. NASF are now called multidisciplinary teams in PHC, which work in a complementary and integrated manner with FHT. Co-financed by the Federal Government, this new arrangement maintains similarities with the work of NASF, but broadens the scope of individual and territorial actions, including care, matrix support, continuing education actions and telehealth services. The composition of multidisciplinary teams includes a broad range of professionals, including up to 11 professionals from different disciplinary backgrounds and eleven medical specialties, with priority given to professionals such as social workers. The integration of diverse areas of competence in collaborative work strengthens co-responsibility in care and enhances the capacity of PHC to address health needs and deliver effective solutions, ensuring the provision of quality care tailored to the population’s needs. Given that multidisciplinary teams are a recent initiative, it is still too early to evaluate their results.

### Brazilian National Program to Improve Access and Quality in Primary Care

PMAQ-AB was a milestone in the institutionalization of the evaluation of PHC in the SUS, linking significant additional funding to team performance and inducing comparable quality standards at the national, regional, and local levels. PMAQ-AB was a Federal Government initiative introduced during the Dilma Rousseff administration, and a new component of the variable PAB was created by the 2011 PNAB. Divided into cycles, the program involved adherence and performance-based agreements, self-assessment, indicator monitoring, external evaluation and renewal of performance-based agreements aimed at addressing shortcomings.

External evaluations were conducted by more than 40 public universities throughout the country in collaboration with the technical team of the Brazilian Ministry of Health’s Primary Care Department, leading to the development of evaluation tools, organization of fieldwork, questionnaire administration in Brazil’s 5,570 municipalities and the publication of results that informed initiatives designed to improve access to services and the quality of PHC ^19^ ^,^ ^20^.

The first cycle (2011-2012) was accompanied by a BHU Census, which provided an unprecedented assessment of infrastructure and helped inform specific investments. In subsequent cycles, the scope of the program was expanded to include oral health teams and NASF, with Cycle III (2015-2017) evaluating more than 37,000 teams and interviewing 140,000 patients. In addition to fostering improvements in work organization and continuing education, the external evaluation served as a pedagogical tool, stimulating critical reflection among health professionals and managers ^19^ ^,^ ^20^ ^,^ ^21^.

However, starting in 2017, PMAQ-AB was progressively dismantled as a result of political and institutional changes, finally being terminated in 2019 and replaced by the Previne Brasil program, which reduced evaluation to a limited set of process indicators and was discontinued in 2024. While the sustainability of the program was undermined by wrangling over the SUS care model and funding, PMAQ-AB remains a benchmark for cooperation across different levels of government and the evaluation of the complexities of PHC ^19^ ^,^ ^20^ ^,^ ^21^.

### More Doctors Program: emergency staffing, medical education and improvements in infrastructure

Created in 2013, the PMM sought to address the shortage and poor distribution of doctors in the SUS through providing emergency staffing in underserved areas, training of general practitioners and investment in infrastructure. The staffing strategy (the More Doctors for Brazil Project - PMMB, acronym in Portuguese) was made possible largely by a cooperation agreement between the Brazilian government, Pan-American Health Organization (PAHO) and Cuban government, which resulted in the hiring of more than 18,000 doctors across 4,500 municipalities at the height of the program (2016), mostly in extremely poor regions, rural areas and urban peripheries. More than 35,000 doctors were hired between 2013 and 2017, 72.8% of whom were non-Brazilian, the vast majority of whom Cuban ^22^ ^,^ ^23^ ^,^ ^24^.

While criticized by medical bodies, which denied the existence of shortages and disparities in the distribution of doctors across the country, the PMMB improved access, reduced regional inequalities and strengthened health team staff retention, although permanent staffing challenges remained. Together with the BHU Upgrade program, which supported upgrades of 26,000 BHU and the construction of new health centers, and PMAQ-AB, the PMM strengthened PHC across multiple dimensions ^21^ ^,^ ^22^ ^,^ ^23^ ^,^ ^24^ ^,^ ^25^ ^,^ ^26^.

In addition to staffing, the PMM promoted the expansion of medical courses (70 new courses between 2013 and 2016) and Family and Community Medicine Residency programs, resulting in significant, albeit still insufficient, growth in the number of specialists in this area. In 2024, Brazil had 15,542 medical specialists and 3,079 Family and Community Medicine residents ^27^, and 29% of BHU had Family and Community Medicine specialists (Table 2).

**Table 2:** Overview of primary health care (PHC) in the Brazilian Unified National Health System (SUS): number and distribution of basic health units (BHU) according to selected characteristics. Brazil and regions, 2024.

Variables	Brazil (%)	North (%)	Northeast (%)	Southeast (%)	South (%)	Central-West (%)
BHU and teams						
Number of BHU (n)	44,938	4,096	17,735	13,375	6,607	3,123
BHU in urban areas	67	55	52	82	79	84
BHU in rural areas or riverine	33	46	48	18	21	16
BHU facilities belonging to the Municipal Health Department	85	91	85	82	90	88
BHU with one FHT	67	54	79	59	61	67
BHU three or more FHT	10	12	7	14	10	14
BHU with OHT	75	63	80	71	70	79
BHU with multidisciplinary PHC support team	42	32	47	46	31	32
BHU with Family and Community Medicine specialist	29	27	25	33	32	28
BHU with doctors from the PMM	43	56	43	38	46	38
BHU with at least one permanently contracted doctor	33	24	23	41	49	33
BHU with private human resource outsourcers *	20	6	19	25	21	20
Provision of PHC services						
BHU that provide medical and nurse consultation	99	94	99	99	99	99
BHU that provide dental consultation	89	79	92	86	88	89
BHU that perform vaccination	85	81	93	80	76	80
BHU that dispense medicines	70	79	83	54	71	56
Community orientation and public participation						
BHU with defined catchment area	85	77	84	89	89	83
BHU that perform community appraisals	66	59	68	68	67	62
BHU that perform annual community appraisals	49	56	51	48	41	43
BHU with an active local health council	36	37	30	42	40	39
Coordination and integration across the regional network						
Use of electronic health records in BHU	87	65	84	92	98	95
Electronic health records shared with other BHU	70	42	64	76	85	83
Electronic health records shared with specialist public services	19	6	8	29	36	20
Frequent communication between teams and specialists	41	42	37	46	43	41
BHU receives the hospital discharge summary always or almost always	28	20	21	42	27	19
BHU schedules the specialist appointments/examinations using the scheduling system and communicates the date to patients	59	52	46	73	71	53
BHU maintains a record/list of patients referred to other services within the health network	57	52	51	68	58	46
BHU tracks referred patient wait times	34	31	30	43	36	25
BHU provides specialist care for acute coronary syndrome	57	44	49	66	71	58
BHU provides specialist stroke care services	61	50	54	68	72	62

FHT: family health teams; OHT: oral health teams; PMM: More Doctors Programs.

Source: based on data from the 2024 BHU Census ^43^ ^,^ ^44^.

Note: % in relative frequency.

* Private human resource outsourcers: Social Health Organizations (OSS, acronym in Portuguese), Civil Society Prganizations of Public Interest (OSCIP), charities, non-governmental organizations (NGOs), private companies and cooperatives.

## Impact of the expansion of FHS coverage on access and population health

A large body of literature ^28^ ^,^ ^29^ ^,^ ^30^ ^,^ ^31^ ^,^ ^32^ ^,^ ^33^ ^,^ ^34^ shows that the expansion of the FHS has promoted consistent improvements in access, availability of a usual source of care and population health, as well as reductions in social and regional inequalities.

The results of the 2019 *Brazilian National Health Survey* (PNS, acronym in Portuguese) confirm that the distribution of the FHS is equitable, with coverage being higher among vulnerable populations, especially low-income and -education households, and reaching 74% in the first income quintile compared to 38% in the highest quintile. Availability of a regular source of care, an indirect indicator of access, was higher among people registered with the FHS (79%) than in those not registered (73%), with 56% these patients using BHU as their regular service ^4^.

Studies show that the FHS has had a significant impact on morbidity and mortality, especially in areas with consistently high coverage: reductions in infant and under-5 mortality ^35^ ^,^ ^36^, and in ambulatory care sensitive conditions among adults and older persons ^37^. The effects were more pronounced in vulnerable groups, contributing to a reduction of regional and social health inequalities ^29^ ^,^ ^38^.

Evidence also shows that the FHS and the Brazilian Income Transfer Program have a synergistic effect, resulting in poverty reduction, improved access and a decline in infant and tuberculosis mortality ^32^ ^,^ ^39^ ^,^ ^40^. Time series studies covering all of Brazil’s municipalities also show that the FHS was associated with a fall in hospital admissions for ambulatory care sensitive conditions ^31^ ^,^ ^34^.

The evidence is mainly derived from longitudinal ecological studies using data from the Brazilian Mortality Information System (SIM, acronym in Portuguese) and Brazilian Hospital Information System (SIH, acronym in Portuguese). More recently, the findings of cohort studies using individual data from the *Cadastro Único* (a unified registry for social programs) linked to health databases suggest a plausible causal link between the FHS and health service utilization and indicators ^36^.

## Setbacks

The development of PHC in Brazil occurred alongside the institutionalization of the SUS, which has been marked by both incremental advances and setbacks. Against a backdrop of institutional rupture, the review of the PNAB in 2017 deprioritized the FHS, which up to that point was the central pillar of PHC, representing a significant turning point ^5^.

Between 2016 and 2022, federal policies implemented by right-wing and far-right governments gave rise to significant setbacks: weakening of multidisciplinarity caused by reductions in the number of CHW; and the elimination of funding for NASF in 2019 and the replacement of the latter with primary health care teams (PHCT). The latter were made up of just one doctor and nurse with 20- or 30-hour contracts, a far cry from the territorial- and community-based approach of the FHS, but with equivalent funding. These measures led to the dismantling of almost three decades of consolidation of the FHS.

Between 2019 and 2023, with the introduction of the Previne Brasil program, the original funding criterion based on affiliation among the catchment area population was replaced by individual registration, limiting collective actions, restricting access to registered patients and undermining the principles of universality, comprehensiveness and equity. This arrangement strengthened the concept of selective PHC based on restricted neoliberal citizenship, focusing on a “minimal SUS” intended for the poor ^5^ and shifting priority to doctor-centered teams (PHCT), undermining the comprehensiveness of care and interrupting the development of the FHS ^5^ ^,^ ^41^.

The withdrawal of Cuban doctors from the PMMB at the end of 2018 illustrated the vulnerability of the emergency staffing model, resulting in serious gaps, especially in rural and remote municipalities ^42^. In 2019, the Doctors for Brazil Program (PMpB, acronym in Portuguese) was launched under the management of the Agency for the Development of Primary Health Care (ADAPS, acronym in Portuguese), a private organization operating as an autonomous social service, sparking criticism because it privatized PHC governance and due to its initial ineffectiveness ^41^. The first PMpB selection process was only launched in 2022, with limited coverage, and PMMB selection processes were therefore renewed amid the health crisis caused by the COVID-19 pandemic.

## Current overview of the FHS

Over three decades, continuous federal financial incentives have driven the expansion of the FHS throughout the country. In 2024, with 54,600 FHS teams, 300,000 CHW and 32,000 OHT, the potential population coverage of the FHS was estimated at 78% of the national population, with official data suggesting 100% PHC coverage (Table 1).

However, this expansion has been geographically uneven, especially when comparing urban and rural areas. While 88% of the country’s BHU have FHT, 67% have only one team, 11% have only two teams and 10% have three or more teams. BHU with only one team predominate in small municipalities, while BHU with two or more teams tend to be concentrated in larger municipalities ^43^ (Table 2). Facilities with a larger number of teams generally have a wider range of equipment, support services and other professionals with greater capacity to address health needs and deliver effective solutions. This unevenness adversely affects equity. The fact that around 70% of municipalities in Brazil have fewer than 20,000 inhabitants requires tailored arrangements to promote greater capacity to address health needs and deliver effective solutions in PHC even in smaller locations, despite losing economies of scale.

BHU offer comprehensive services throughout the life cycle (child health, maternal health, women’s health, older adult health), including health promotion and disease prevention, responding to acute health problems and caring for patients with chronic conditions (hypertension, diabetes, tuberculosis), among others. However, the scope of services varies across regions, being broader in urban centers, although rural doctors often perform more procedures ^44^ ^,^ ^45^ ^,^ ^46^.

Territorialization and the community-oriented approach, which form the foundation of the FHS, still require further consolidation: 85% of BHU have a designated catchment area, with only half conducting annual community assessments and just over a third maintaining active local health councils ^44^ (Table 2). Community action and annual participatory community appraisals help gain a greater understanding of the territory, potential resources, gaps in services and difficulties accessing health promotion actions. They therefore provide the basis for planning actions to address the needs of the community, based on up-to-date data on local historical, cultural, social, environmental and health characteristics and risks and vulnerabilities.

However, community action is characterized by regional inequalities and weaknesses, achieving greater reach in the Northeast, as shown by the response to the COVID-19 pandemic in this region ^47^. Community action is intimately linked to the work of CHW in homes and the community, which has been scaled back in many places due to the heavy administrative workload of these workers in BHU ^48^.

BHU are the first point of contact and primary gateway to the health care system, acting as a gatekeeper and important filter to specialist services by coordinating care. However, integration with the specialist and hospital care networks faces structural barriers, especially in the North and Northeast, where the shortage of public services and concentration of resources in urban areas exacerbate historical inequalities.

Starting in 2023, the Lula administration, under the leadership of Health minister Nísia Trindade, began to rebuild the SUS, reemphasizing FHS as the priority PHC mode. Funding for multidisciplinary care was resumed, focusing on multidisciplinary teams, and the PMMB was reformulated and expanded, placing greater emphasis on the training of PHC specialists and staff retention, with contracts lasting up to eight years and new financial incentives. In 2025, 25,000 doctors were working in 43% of the country’s BHU across 80% of the country’s municipalities through the PMMB, with the project playing a pivotal role in strengthening PHC and reducing health inequalities. In addition, the government resumed the accreditation of new oral health teams, which had been suspended, distinguishing PHC in Brazil from other health systems on the global stage.

Multidisciplinary teams are currently attached to different types of PHC teams and have a broader range of professionals, including new medical specialties and telehealth professionals. Despite this new arrangement and the fact that municipal governments have autonomy over the definition of team composition, the coverage of multidisciplinary teams remains limited and the consolidation of interprofessional practices and ensuring systematic support for BHU remains a challenge.

## Ongoing challenges

Despite renewed emphasis on the FHS model since 2023, structural problems remain that have yet to be addressed by incremental reforms. Challenges include the following: provision of adequate funding to ensure universal coverage of the FHS with the provision of quality care; competition for public resources and risks posed by commodification in PHC; the provision of adequate health workforce training and retention of PHC health workers; improving health equity, with a focus on addressing structural racism; and care coordination and integration into the wider care network to ensure comprehensive care.

### Funding: ensuring sufficient resources to enable universal access to quality primary health care

Achieving universal coverage of the FHS with the provision of quality care requires sufficient resources to fully equip BHU and ensure competent multidisciplinary teams with suitable staffing for the size of the catchment area and capacity to address health needs and deliver effective solutions. PHC funding, which should be a tripartite responsibility, is provided primarily by municipalities due to Federal Government fiscal austerity policies and the limited contribution of state governments, exacerbating intergovernmental conflicts.

The new federal co-financing model introduced in 2024, which could be called “More Family Health” restored priority to the FHS, increasing fixed team funding and incentives linked to the level of social vulnerability of the catchment population. The model also incorporates quality indicators agreed via tripartite negotiations, reaffirming the need to align funding to a care model based on territorialization, multidisciplinarity and integration into the wider health network. However, the coexistence of more than 6,000 PHCT (Table 1) and programs promoting access to specialties - which are necessary but still lack effective integration - places a strain on the system and highlights conflicts between different care models.

In addition, with health care expenditure accounting for less than 4% of gross domestic product (GDP) - which is far below the proportion spent by other countries with universal systems (around 7%) - chronic underfunding of the SUS remains a major obstacle to the expansion and improvement of PHC. As argued in the Lancet Global Health Commission report on financing primary health care, countries should invest more and invest better in PHC ^49^. However, our perspective differs significantly from the narrow, selective approach advocated in the document for low- and middle-income countries, where universal coverage is limited to a basic package of health services, thereby restricting the expansion of other service areas ^49^.

To achieve universal FHS coverage in accordance with the guiding principles of the SUS (universality, comprehensiveness and equity), the fiscal framework needs to be reviewed to provide sufficient constitutional funding for the SUS, guaranteeing that federal transfers for PHC to municipalities meet local needs ^50^. Investment is also needed to improve facilities, clinical equipment, digital and telehealth and to promote continuing education and professional development in order to strengthen the capacity of PHC to address health needs and deliver effective solutions, reduce unnecessary referrals and ensure the effective coordination of care with specialist services.

### Commodification in PHC

PHC, which has historically been less affected by commodification within the SUS, has seen increasing involvement of the private sector in service provision ^51^. While most BHU remain under municipal ownership (85%), management by Social Health Organizations (OSS, acronym in Portuguese), Civil Society Prganizations of Public Interest (OSCIP, acronym in Portuguese) and charities has expanded, especially in large urban centers. The 2024 BHU Census shows that 20% of centers are operated under private management arrangements, signaling the expansion of private sector involvement in primary care (Table 2).

Furthermore, the creation of the Brazilian SUS Management Support Agency (AgSUS, acronym in Portuguese) - an autonomous social service established to replace the ADAPS and provide operational support for the implementation of health policy, particularly in the areas of Indigenous Health and PHC - has opened up further possibilities for private outsourcing at the federal level, exacerbating uncertainties regarding the maintenance of PHC as a public good.

This move towards outsourcing and public-private partnerships - even those involving nonprofit organizations - appears to place PHC services within a broader trend towards the commodification of health, which involves the transfer of public funds to private entities, with potential impacts on the public nature of the SUS ^52^.

### FHS workforce: training, employment relationship and career paths

The consolidation of the FHS requires the provision of qualified health workers with employment stability and long-term retention, which is essential for continuity of care. Since the 1990s, continuing education and training policies have sought to strengthen PHC practices. However, initiatives have experienced discontinuities and high staff turnover, and integration between teaching, services and the community is poor ^53^.

Historically, general practitioner training in medical schools has been limited, with Family and Community Medicine only being recognized as a specialty in 2002 and not being adequately valued professionally, which is reflected in poor working terms and conditions and unequal pay.

However, there has been an expansion of multidisciplinary training for PHC over time, including specializations and Family and Community Nursing, Family and Community Medicine and multidisciplinary residency programs, as well as professional master’s programs at public universities, such as ProfSaúde (family health professional master’s degree), funded by the Brazilian Ministry of Health and the Brazilian Ministry of Education. The latter was originally aimed solely at physicians but now includes other professions.

Multidisciplinary training initiatives are aimed at promoting interprofessional collaboration. By integrating decision-making and responsibilities, interprofessional collaboration constitutes a central strategy for addressing the complexities of multimorbidity and comprehensive care, with emphasis on broadening the scope of nursing and advanced person-centered practices ^54^. However, the institutionalization of these practices faces structural barriers, such as the hegemony of the doctor-centered logic and gender, racial and class hierarchies ^55^.

Staff retention remains a critical challenge that needs to be addressed to improve the quality of PHC. In 2024, only 33% of BHU had at least one permanently contracted doctor, and 42% had doctors employed under the PMM, with temporary contracts and no job stability. The persistence of poor working terms and conditions hampers the continuity of care and weakens the healthcare model. The absence of a civil service career path for PHC professionals SUS workers is the result of financial constraints, labor and social security reforms and the Fiscal Responsibility Law, which limit employment stability ^56^.

Thus, strengthening the FHS requires consistent continuing education and development policies and the provision of stable employment through the establishment of civil service health career paths and longer tenures for health team members, with a view to promoting interprofessional collaboration as part of a national initiative to strengthen the SUS.

### Health equity and addressing structural racism in PHC

Structural racism is a key determinant of health inequalities in Brazil, which disproportionately affect Black and Mixed-race people, and displays pronounced intersections with gender and social class ^57^. Evidence suggests that discrimination in health care services creates economic, organizational and cultural barriers, limiting timely access to quality care ^58^.

The creation of the Brazilian National Policy on Comprehensive Healthcare for the Black Population (PNSIPN, acronym in Portuguese), published in 2007 and reviewed in 2017, marked a milestone for the recognition of ethnic and racial inequalities and institutional racism as social determinants of health ^59^ ^,^ ^60^. However, policy implementation remains limited: in 2024, only 55% of BHU reported adopting strategies to address racism, and only 13% had provided specific training on the policy in the past year ^43^.

The territorial and community-based approach adopted by the FHS means the strategy is uniquely positioned to promote equitable access to comprehensive care, strengthen coordination with social policies and incorporate intersectoral practices aimed at addressing racial inequalities. However, the effectiveness of the FHS depends on continuing health worker training, the institutionalization of the PNSIPN and the integration of health actions and social protection and gender, class and race/ethnicity equity policies and initiatives ^58^.

The development of tailored and intersectional policies is essential to reduce inequalities and consolidate the SUS as a universal system that delivers comprehensive care and promotes social justice ^58^.

### Coordination, integration into the wider care network and continuity of care

The provision of quality PHC requires effective care coordination, integration into regional SUS networks and continuity of care. The 2023 Brazilian National Policy on Specialized Health Care (PNAES, acronym in Portuguese) reaffirms that PHC is the primary point of entry to the health system and functions as the central communication hub of the Health Care Network (RAS, acronym in Portuguese), playing a pivotal role in organizing access to care, focusing on strengthening clinical capacity and shared responsibility for care ^61^.

The literature highlights that PHC is widely recognized as a key component of all high-performing health systems ^49^. However, barriers to coordination persist in Brazil, stemming from the fragmentation of care, the shortage of specialized services, and shortcomings in interprofessional collaboration and information management and coordination.

Continuity of quality care requires information continuity, which begins at the BHU with the use of electronic health records, which has witnessed significant expansion in PHC over the last decade. The use of electronic health records in BHU rose from 14% in 2012 to 39% in 2018, during the third cycle of the PMAQ-AB ^19^, and reaching 89% by 2024. Sharing of electronic health records between BHU is frequent (70%); however, less than one-fifth (18%) of BHU shared records with specialized public services in 2024. Lack of interoperability and the fact that less than one-third of BHU receive patient hospital discharge summaries undermines information continuity. Furthermore, communication between FHS teams and specialists is frequent in only 41% of BHU (Table 2).

Scheduling mechanisms also display weaknesses, with only 57% of BHU recording patient referrals and 34% monitoring waiting times. In 44% of BHU, patients receive a paper form to request an appointment themselves, showing that the accountability of PHC for specialized care is limited (Table 2).

While coverage of referral services for breast and cervical cancer, high-risk pregnancies, childbirth and mental health is greater than 85%, critical gaps in the definition of referral services remain for cardiovascular diseases in regions such as the North and Northeast ^43^.

To strengthen PHC’s function as coordinator of the RAS it is necessary to: broaden the clinical scope of FHS teams and enhance their capacity to address health needs and deliver effective solutions; promote operational integration between BHU and scheduling centers to reduce wait times and optimize patient care pathways; ensure full interoperability between electronic health records and other information systems; foster ongoing interprofessional communication; provide a clear definition of regional patient flows and referral services, increasing the availability of public specialized care services and strengthening PHC’s accountability for referrals and continuity of care ^62^.

## Final considerations

Advances in population coverage are overshadowed by gaps in care and the inability to ensure the full implementation of the FHS model aimed at providing community-oriented territory-based care integrated into the wider care network. The pillars of this comprehensive approach are: multidisciplinary teams that foster interprofessional collaboration; a territory- and community-based focus; health surveillance and accountability for the health of the population in a given territory; public participation and intersectoral collaboration; expanded scope of individual and collective actions; care coordination and integration into the regional network; interculturality; and recognition of the value of the PHC workforce.

There is an urgent need to close gaps in care, incorporate technology to enhance the capacity of PHC to address health needs and deliver effective solutions, improve network organization and integration, and ensure adequate and sustainable funding based on criteria that promote equity and quality and address population health needs ^62^. Only through systemic coordination among PHC, specialized services and hospitals within equitable regional networks will it be possible to overcome fragmentation and consolidate the SUS as a universal system that delivers effective comprehensive care ^62^.

The full implementation of the FHS care model requires a renewed commitment to the underlying principles of public participation, the territory- and community-based approach, integration of health surveillance and timely individual care, health promotion and disease prevention, network integration, and intersectoral approaches to promote health and reduce gender, race and ethnic, and social inequalities.

Without doubt, to consolidate the FHS it is necessary to overcome institutional vulnerability to political change and uphold the underlying principles of the SUS embodied in the Constitution as the non-negotiable guiding principles of PHC policy.

## Data Availability

The sources of information used in the study are indicated in the body of the article.
