# Supplementary material for: Thirty years of the Family Health Strategy in the Brazilian Unified National Health System: milestones, advances, setbacks and challenges
Source: Cad Saude Publica. 2026 Jun 22;42:e00206025. doi: 10.1590/0102-311XEN206025 (PMC13286402; doi:10.1590/0102-311XEN206025)
Supplement: Translation

# Translation: 30 anos da Estratégia Saúde da Família no Sistema Único de Saúde: marcos, avanços, retrocessos e desafios

**DOI:** 10.1590/0102-311XPT206025

**Authors:** Ligia Giovanella, Maria Helena Magalhães de Mendonça, Estela Márcia Saraiva Campos, Ana Luiza Queiroz Vilasbôas, Rosana Aquino, Luiz Augusto Facchini

**Affiliations:** 1 Centro de Estudos Estratégicos da Fiocruz Antonio Ivo de Carvalho, Fundação Oswaldo Cruz, Rio de Janeiro, Brasil.; 2 Escola Nacional de Saúde Pública Sergio Arouca, Fundação Oswaldo Cruz, Rio de Janeiro, Brasil.; 3 Faculdade de Medicina, Universidade Federal de Juiz de Fora, Juiz de Fora, Brasil.; 4 Instituto de Saúde Coletiva, Universidade Federal da Bahia, Salvador, Brasil.; 5 Departamento de Medicina Social, Universidade Federal de Pelotas, Pelotas, Brasil.

**Keywords:** Estratégia Saúde da Família, Atenção Primária à Saúde, Sistema Único de Saúde

## Abstract

Ensaio sobre a trajetória e desafios persistentes da Estratégia Saúde da Família (ESF) no Brasil, a partir de inquietações quanto às perspectivas para plena implementação desta abordagem de atenção primária à saúde de base territorial e orientação comunitária no Sistema Único de Saúde (SUS). Examina a trajetória de expansão e revisita criticamente os principais marcos institucionais da ESF nestes 30 anos, destacando a indução financeira federal associada ao processo de municipalização; ampliação da mutiprofissionalidade no trabalho desenvolvido nas unidades básicas de saúde (UBS) e territórios; indução da qualidade articulada à avaliação da estrutura e processo das equipes e transferências financeiras associadas ao desempenho; e provimento emergencial de médicos em áreas desassistidas, associado à formação profissional. Sintetiza repercussões da expansão da cobertura da ESF e da adoção de práticas centradas em pessoas, famílias e comunidades sobre a saúde e o acesso. Retrocessos, entre 2016 e 2022, com restrições à multiprofissionalidade abordagem comunitária e ameaças à universalidade e integralidade são abordados criticamente, bem como a retomada, pelo Governo Federal, da prioridade para a ESF a partir de 2023. Dados do Censo Nacional das UBS 2024 ilustram a situação atual resultante desta trajetória. Na análise dos desafios para a efetiva mudança no modelo assistencial e universalização da ESF, destaca-se suficiência do financiamento; estabilidade funcional, formação e trabalho colaborativo interprofissional; os riscos de mercantilização na atenção primária à saúde (APS); coordenação do cuidado e rol da APS na regulação assistencial e; as desigualdades geográficas, sociais e étnico-raciais no acesso.

## Introdução

A atenção primária à saúde (APS) no Sistema Único de Saúde (SUS), denominada atenção básica na política nacional em contraposição à abordagem seletiva e focalizada ^1^, teve um salto qualitativo a partir de 1994 com a implementação gradual do Programa Saúde da Família, atual Estratégia Saúde da Família (ESF).

Esse processo marcou uma inflexão no modelo assistencial, com orientação comunitária, territorializada, integrada à rede e equipe multiprofissional. Múltiplas evidências indicam que a ESF alcançou ampla cobertura populacional, melhorou o acesso e os indicadores de saúde, reduzindo desigualdades ^2^ ^,^ ^3^ ^,^ ^4^.

Apesar dos avanços, a ESF não se desenvolveu em sua plenitude, condicionada por barreiras financeiras, de infraestrutura e estabilidade na composição das equipes. Entre 2016 e 2022, o cenário político e institucional intensificou fragilidades históricas, comprometendo a universalidade, a equidade e a integralidade do SUS ^5^.

Na celebração dos 30 anos da ESF, este ensaio parte da inquietação sobre o futuro e perspectivas deste modelo inovador em APS, reconhecido globalmente ^6^, e pergunta-se sobre os principais desafios para sua plena implementação. Para tal, apresenta uma análise crítica e revisita seus principais marcos históricos e institucionais no processo de expansão desde 1994 com a incorporação da saúde bucal, a criação dos Núcleos de Apoio à Saúde da Família (NASF; posteriormente Núcleos Ampliados de Saúde da Família), o Programa de Melhoria do Acesso e da Qualidade da Atenção Básica (PMAQ-AB) e o Programa Mais Médicos (PMM). Em continuação, discute-se as repercussões da expansão da cobertura da ESF e da adoção de práticas centradas nas pessoas, famílias e comunidades na saúde da população e no acesso. Retrocessos, entre 2016 e 2022, são abordados criticamente, bem como a retomada, pelo Governo Federal, da prioridade para a ESF a partir de 2023. A situação atual da APS brasileira é completada com informações recentes do Censo Nacional das Unidades Básicas de Saúde (Censo das UBS) 2024 que ilustram também os desafios destacados.

Por fim, examina-se os desafios persistentes para a consolidação do modelo ESF, incluindo o financiamento adequado e suficiente para a universalização da ESF, os processos de mercantilização na APS, o fortalecimento da formação e da educação permanente, os vínculos estáveis e as carreiras em saúde, coordenação, continuidade do cuidado e integração da APS às redes de atenção e a redução de desigualdades territoriais, sociais e étnico-raciais.

O artigo busca contribuir para o debate sobre o futuro da ESF, fomentando a necessidade de seu fortalecimento como estratégia central para efetivar os princípios do SUS diante das transformações demográficas, epidemiológicas e sociais contemporâneas.

## Trajetória da atenção primária à saúde no SUS

Com a criação do SUS em 1988, sob o princípio organizativo da descentralização, os municípios gradualmente assumiram a responsabilidade pela APS com prestação de serviços de atenção básica e mudanças no modelo assistencial promovidas por normativas e incentivos federais e iniciativas locais. Neste processo de expansão, constitui-se ampla rede capilarizada de unidades básicas de saúde (UBS) por todo o país. Em 2024, esta rede somava aproximadamente 45 mil UBS distribuídas nos 5.570 municípios brasileiros, assegurando acesso universal e gratuito.

A seguir, são descritos e analisados os principais marcos históricos-institucionais dessa trajetória.

### O Programa Saúde da Família e sua transformação em estratégia

Implantado em 1994, o Programa Saúde da Família introduziu uma abordagem de APS de base comunitária e territorial, integrada à rede no SUS, com equipes multiprofissionais voltadas ao cuidado continuado de indivíduos, famílias e comunidades, como estratégias de fortalecimento do vínculo com usuários, com destaque para o papel central dos ACS.

Originalmente, as equipes eram formadas por um médico com atuação generalista, um enfermeiro, um técnico ou auxiliar de enfermagem e quatro a seis agentes comunitários de saúde (ACS), selecionados entre os moradores da comunidade. Os ACS têm papel fundamental na ação comunitária no território em visitas domiciliares rotineiras a cerca de 150 famílias residentes em sua microárea. Os ACS realizam o cadastramento das famílias, o diagnóstico comunitário e as ações de proteção à saúde, identificando grupos de risco ^7^.

Portanto, as equipes também são responsáveis por ações de prevenção de agravos, vigilância e promoção da saúde, com ênfase na determinação social do processo saúde e adoecimento, que inclui explicitamente as ações intersetoriais e a participação social como parte de intervenções implementadas segundo peculiaridades dos territórios e das características intrínsecas de gestores, profissionais e usuários ^8^.

Com a *Norma Operacional Básica nº 01/1996*, o Programa Saúde da Família tornou-se estratégia de organização da APS, financiada por incentivo federal específico, com vistas a responder aos princípios de universalidade e equidade no acesso aos serviços de saúde a toda população brasileira. O município tornou-se o protagonista da implantação e gestão da APS, cujos serviços devem estar o mais próximo possível de onde as pessoas vivem ^9^.

As transferências federais desempenharam papel decisivo na indução da ESF e no processo de descentralização do SUS. O Piso de Atenção Básica (PAB) fixo assegurou autonomia municipal, enquanto o PAB variável fomentou a difusão das equipes de ESF, consolidando o modelo comunitário e territorial, integrado à rede, com efeitos positivos na saúde da população. Em 2006, a Política Nacional de Atenção Básica (PNAB) consolidou o Programa Saúde da Família como ESF, reafirmando seu caráter prioritário e substitutivo em relação ao modelo tradicional de atenção básica ^10^ ^,^ ^11^ ^,^ ^12^.

Inicialmente seletivo e focalizado, visando expandir a cobertura de APS em áreas desassistidas, o PSF alcançou progressivamente os grandes centros urbanos. Nas grandes cidades, especialmente do Centro-Sul do país, a estratégia envolveu a conversão de UBS tradicionais ao novo modelo ESF, sem implicar necessariamente em aumento da cobertura populacional ^2^ ^,^ ^7^ ^,^ ^13^ ^,^ ^14^. Entre 2003 e 2009, esse processo foi incentivado pelo Projeto de Expansão e Consolidação do Saúde da Família (PROESF), cujos estudos de linha de base foram fundamentais para a institucionalização do monitoramento e da avaliação da APS, concomitante à criação da Coordenação de Acompanhamento e Avaliação da Atenção Básica no Departamento da Atenção Básica da Secretaria de Atenção à Saúde do Ministério da Saúde ^14^.

As versões da PNAB em 2006, 2011 e 2017 introduziram ajustes no escopo das equipes e parâmetros de cobertura. A PNAB 2011 expandiu a saúde da família incluindo modalidades específicas para populações vulneráveis por meio de equipes ribeirinhas, UBS Fluviais e Consultórios de Rua, adotou a APS como sinônimo de atenção básica, e reduziu o contingente populacional recomendado por equipe de 4 mil para em média 3 mil pessoas, ou menos em populações mais vulnerabilizadas. A PNAB 2017 aumentou o parâmetro de referência para em média 3.500 pessoas e, infelizmente, excluiu o incentivo financeiro exclusivo para a ESF ^5^.

Ainda assim, a ESF consolidou-se como eixo estruturante da APS no Brasil, mas sua implementação heterogênea e a persistência de desafios estruturais evidenciam a necessidade de maior qualificação, financiamento estável e integração em rede para assegurar sua plena efetividade ^7^. A evolução da cobertura e os principais marcos da trajetória da ESF são apresentados na Figura 1 e na Tabela 1.

**Figura 1:** Linha do tempo com principais marcos históricos da Política Nacional de Atenção Básica (PNAB) e evolução do número de equipes de saúde da família (eSF), da cobertura populacional estimada de equipes de atenção primária à saúde (eAP) e de saúde bucal (eSB) na atenção primária à saúde (APS), segundo a Secretaria de Atenção Primária à Saúde, Ministério da Saúde. Brasil, 1998 a 2024.

**Tabela 1:** Evolução da cobertura populacional * e número de equipes da Estratégia Saúde da Família (ESF), equipes de saúde bucal (eSB), Núcleos Ampliados de Saúde da Família (NASF) e agentes comunitários de saúde (ACS). Brasil, 1998 a 2024.

Ano	eSF (n)	eAP (n)	Cobertura populacional estimada pelas equipes da ESF (%)	Cobertura da APS (%)	eSB (n)	Cobertura populacional estimada por eSB vinculadas à eSF (%)	Cobertura populacional estimada pela saúde bucal na APS (%)	NASF (n)	ACS (n)
1998	3.062	-	6,6	-	0	-	-	0	-
1999	4.114	-	8,8	-	0	-	-	0	-
2000	8.503	-	17,6	-	0	-	-	0	-
2001	13.155	-	25,6	-	2.248	-	-	0	-
2002	16.698	-	32,1	-	4.261	-	-	0	-
2003	19.068	-	35,7	-	6.170	-	-	0	-
2004	21.232	-	39,9	-	8.951	-	-	0	-
2005	24.564	-	42,8	-	12.603	-	-	0	-
2006	26.729	-	46,2	-	15.086	-	-	0	-
2007	28.195	-	48,0	60,3	17.508	29,9	46,2	0	245.663
2008	29.769	-	51,0	64,3	19.280	33,3	49,5	0	257.578
2009	30.898	-	51,4	65,0	20.626	34,6	49,8	0	261.839
2010	32.243	-	53,0	66,2	21.999	36,5	50,8	1.317	270.775
2011	33.135	-	54,6	67,1	23.076	38,4	52,7	1.564	275.246
2012	33.899	-	55,5	68,3	23.586	38,9	53,0	1.929	283.404
2013	35.567	-	57,5	70,6	24.131	39,4	52,9	2.767	284.136
2014	39.426	-	61,1	73,1	25.327	39,8	52,5	3.898	288.335
2015	40.765	-	62,5	73,7	25.891	40,3	52,1	4.288	285.736
2016	41.061	-	62,6	74,1	25.827	39,9	51,7	4.406	286.556
2017	42.513	-	63,9	74,6	27.082	41,2	51,9	4.886	284.867
2018	43.016	-	64,2	74,6	28.050	42,1	52,7	5.517	285.570
2019	43.755	-	64,5	74,8	28.991	43,1	53,0	5.487	284.196
2020	43.286	-	63,6	76,1	30.606	45,0	56,1	-	287.064
2021	47.501	3.869	-	69,5	29.816	46,1	56,6	-	288.771
2022	48.817	3.712	-	75,1	28.054	-	40,4	-	291.378
2023	51.369	5.708	-	79,7	31.035	-	44,8	-	295.406
2024	54.629	6.355	-	100,0	34.591	-	45,9	4.567 **	300.104

APS: atençao primária à saúde; eSF: equipes de saúde da família.

Fonte: e-Gestor Atenção Primária/Ministério da Saúde (https://egestoraps.saude.gov.br/), considerando relatórios públicos de histórico do quantitativo de equipes e serviços custeados, cobertura da atenção básica, cobertura da APS, cobertura de saúde bucal, financiamento da APS e cobertura de ACS, conforme disponibilidade dos indicadores e metodologia vigente em cada período analisado (1998-2024).

* O indicador “Cobertura populacional estimada pelas equipes da ESF” foi descontinuado após 2020. A partir de 2021, o indicador “Cobertura da APS” passou a ser calculado com base na população cadastrada pelas eSF e eAP financiadas pelo Ministério da Saúde. Em 2024, passou-se a utilizar o indicador “Cobertura potencial da APS” que considera todos os tipos de equipes cofinanciadas pelo Ministério da Saúde e as equipes financiadas pelos estados e municípios. Em 2024, o indicador “Cobertura populacional estimada pela saúde bucal na APS” adotou novo método, considerando a cobertura populacional da Estratégia Saúde Bucal vinculada exclusivamente à ESF, incluindo equipes de Consultório na Rua (eCR), equipes de saúde da família ribeirinha (eSFR) e equipes de atenção primária prisional (eAPP);

** Considera a retomada do financiamento federal das equipes multiprofissionais na APS, no modelo de equipes multiprofissionais (eMulti).

### O Brasil Sorridente e as equipes de saúde bucal

A partir de 2000, as equipes de saúde bucal (eSB) foram progressivamente incorporadas à ESF, com duas modalidades, uma composta por cirurgião-dentista e auxiliar em saúde bucal e outra que também inclui um técnico em saúde bucal. Em 2004, o primeiro governo Luiz Inácio Lula da Silva lançou o Programa Brasil Sorridente, como base para a Política Nacional de Saúde Bucal (PNSB), visando a ampliação do acesso e a integralidade do cuidado, por meio de ações individuais e coletivas, de promoção, prevenção, diagnóstico, tratamento e reabilitação, incluindo a ampliação da fluoretação da água e a vigilância em saúde bucal no SUS ^15^.

A PNSB também estruturou a rede especializada com a criação dos Centros de Especialidades Odontológicas (CEO), Laboratórios Regionais de Próteses Dentárias (LRPD) e, mais recentemente, os Serviços de Especialidades em Saúde Bucal (Sesb), voltados a municípios de pequeno porte.

As diretrizes políticas e os incentivos financeiros federais promoveram mudanças expressivas no modelo assistencial e maior inserção das práticas odontológicas na APS. Em 2004 eram 8,9 mil eSB cofinanciadas pelo Ministério da Saúde (Tabela 1) e em 2008 já totalizavam 19.280 equipes, com importante diminuição de pessoas que nunca tinham consultado um dentista no país ^15^.

Apesar da redução significativa de barreiras de acesso, a cobertura das eSB permaneceu inferior à das equipes de saúde da família (eSF) e apresentou estagnação entre 2019 e 2022 (Tabela 1), com retrocessos nas ações ofertadas ^16^ (Tabela 1 e Figura 1).

A institucionalização da PNSB pela *Lei nº 14.572/2023* ^17^ representa um marco para sua sustentabilidade como política permanente, destacando o SUS na comparação com países com sistemas universais de saúde, que em geral não contemplam políticas ampliadas de saúde bucal. Ainda assim, persistem desafios relacionados ao fortalecimento do trabalho interprofissional e ao equilíbrio entre demandas individuais e ações coletivas de promoção da saúde.

Para tanto, será crucial investir em inovações tecnológicas, como prontuários eletrônicos integrados, teleodontologia e ferramentas digitais para monitoramento epidemiológico, ampliando a capacidade resolutiva das equipes e a coordenação do cuidado com a rede especializada. Também se impõe a universalização da cobertura das eSB em consonância com a ESF, reduzindo desigualdades regionais e sociais ^17^.

### A expansão da multiprofissionalidade: Núcleos Ampliados de Saúde da Família

Lançados em 2008 por meio de incentivo federal, os NASF foram concebidos para ampliar a multiprofissionalidade da ESF, incorporando profissionais de saúde de diferentes áreas e especialidades, como psicólogos, nutricionistas, fisioterapeutas, assistentes sociais, educadores físicos, psiquiatras, entre outros e fortalecendo o apoio matricial às equipes de atenção básica. Seu propósito foi diversificar o escopo de ações, ampliar a capacidade resolutiva das UBS e promover práticas interprofissionais por meio do cuidado compartilhado, educação permanente e intervenções no território ^18^. Entretanto, a implantação mostrou-se heterogênea, frequentemente reproduzindo modelos ambulatoriais centrados em atendimentos individuais ou funções gerenciais em detrimento da proposta de colaboração integrada ^18^.

Em 2019, os NASF foram desfinanciados pelo Ministério da Saúde em um contexto de ênfase na centralidade do trabalho médico, comprometendo a multiprofissionalidade na APS ^5^. Em 2023, o programa foi reformulado em cenário de reconstrução do SUS e de reiteração da prioridade para a ESF. Agora denominadas de equipes multiprofissionais na APS, as eMulti atuam de maneira complementar e integrada às eSF. O novo arranjo com cofinanciamento federal mantém similaridades com o trabalho do NASF ampliando, contudo, seu escopo de práticas de cuidado individuais e no território com ações assistenciais, de matriciamento e educação permanente, às quais se somam a telessaúde. A composição das eMulti é abrangente, integradas por até onze profissões de diferentes áreas do conhecimento e onze especialidades médicas, com prioridade para inclusão de profissionais como assistentes sociais. A integração das diversas competências em trabalho colaborativo fortalece a corresponsabilidade no cuidado, amplia a capacidade resolutiva da APS, propiciando cuidado mais qualificado e alinhado às necessidades da população. Com processo recente de implantação, seus resultados ainda não podem ser avaliados.

### O Programa de Melhoria do Acesso e Qualidade na Atenção Básica

O PMAQ-AB representou um marco na institucionalização da avaliação da APS no SUS, associando um expressivo financiamento adicional ao desempenho das equipes e induzindo padrões de qualidade comparáveis em nível nacional, regional e local. O PMAQ-AB foi iniciativa da presidenta Dilma Rousseff e o novo componente do PAB variável foi instituído pela PNAB 2011. Estruturado em ciclos, envolvia adesão e contratualização, autoavaliação, monitoramento de indicadores, avaliação externa e recontratualização orientada à superação de insuficiências.

Em colaboração com a equipe técnica do Departamento de Atenção Básica do Ministério da Saúde, a avaliação externa foi conduzida por mais de 40 universidades públicas distribuídas em todo o país, que lideraram a construção dos instrumentos avaliativos, a organização do trabalho de campo, a aplicação dos questionários em 5.570 municípios brasileiros e a publicação de resultados que apoiavam as iniciativas de melhoria do acesso e da qualidade na APS ^19^ ^,^ ^20^.

Seu primeiro ciclo (2011-2012) foi acompanhado de um censo das UBS, que gerou diagnóstico inédito de infraestrutura e subsidiou investimentos específicos. Nos ciclos subsequentes, ampliou a abrangência para eSB e NASF, alcançando no Ciclo III (2015-2017) mais de 37 mil equipes avaliadas e 140 mil usuários entrevistados. Além de induzir melhorias na organização do trabalho e na educação permanente, a avaliação externa funcionou como dispositivo pedagógico ao estimular reflexão crítica entre profissionais e gestores ^19^ ^,^ ^20^ ^,^ ^21^.

A partir de 2017, contudo, o PMAQ-AB foi progressivamente esvaziado em meio a mudanças político-institucionais, sendo encerrado em 2019 e substituído pelo Programa Previne Brasil, que reduziu a avaliação a um conjunto restrito de indicadores de processo, extinto em 2024. O PMAQ-AB permanece como referência de cooperação federativa e de avaliação complexa da APS, ainda que sua sustentabilidade tenha sido comprometida por disputas em torno do modelo de atenção e do financiamento do SUS ^19^ ^,^ ^20^ ^,^ ^21^.

### O Programa Mais Médicos: provimento emergencial, educação médica e melhoria da infraestrutura

Instituído em 2013, o PMM buscou enfrentar a escassez e má distribuição de médicos no SUS por meio de provimento emergencial em áreas desassistidas, formação de médicos generalistas e investimentos em infraestrutura. A estratégia de provimento, denominada de Projeto Mais Médicos para o Brasil (PMMB) foi viabilizada sobretudo por acordo de cooperação com a Organização Pan-Americana da Saúde (OPAS) e o governo de Cuba, assegurando a presença de mais de 18 mil médicos em 4.500 municípios no auge do programa (2016), majoritariamente em regiões de extrema pobreza, áreas rurais e periferias urbanas. Entre 2013 e 2017, mais de 35 mil médicos foram contratados, sendo 72,8% estrangeiros, a grande maioria cubanos ^22^ ^,^ ^23^ ^,^ ^24^.

Embora alvo de críticas de entidades médicas, que negavam a escassez e as disparidades na distribuição dos profissionais no país, o PMMB ampliou o acesso, reduziu iniquidades regionais e garantiu maior regularidade de profissionais nas equipes, ainda que o desafio da fixação estrutural permanecesse. Associado ao Requalifica UBS, que apoiou 26 mil reformas e construções de unidades, e ao PMAQ-AB, o PMM fortaleceu a APS em múltiplas dimensões ^21^ ^,^ ^22^ ^,^ ^23^ ^,^ ^24^ ^,^ ^25^ ^,^ ^26^.

Além do provimento, o PMM promoveu a expansão de cursos de medicina (setenta novos entre 2013 e 2016) e da Residência em Medicina de Família e Comunidade, resultando em crescimento expressivo, embora ainda insuficiente, de especialistas nessa área. Em 2024, o Brasil contava com 15.542 médicos especialistas, 3.079 residentes em Medicina de Família e Comunidade ^27^ e 29% das UBS contavam com especialistas em Medicina de Família e Comunidade (Tabela 2).

**Tabela 2:** Panorama da atenção primária à saúde (APS) no Sistema Único de Saúde (SUS): número de unidades básicas de saúde (UBS) e distribuição das UBS segundo características selecionadas. Brasil e grandes regiões, 2024.

Variáveis	Brasil (%)	Norte (%)	Nordeste (%)	Sudeste (%)	Sul (%)	Centro-oeste (%)
UBS e equipes						
Número de UBS (n)	44.938	4.096	17.735	13.375	6.607	3.123
UBS em área urbana	67	55	52	82	79	84
UBS em área rural ou ribeirinha	33	46	48	18	21	16
UBS em estabelecimento próprio da Secretaria Municipal de Saúde	85	91	85	82	90	88
UBS com uma eSF	67	54	79	59	61	67
UBS com três ou mais eSF	10	12	7	14	10	14
UBS com eSB	75	63	80	71	70	79
UBS com eMulti de apoio à APS	42	32	47	46	31	32
UBS com médico especialista em Medicina de Família e Comunidade	29	27	25	33	32	28
UBS com médicos do PMM	43	56	43	38	46	38
UBS com pelo menos um médico servidor público estatutário	33	24	23	41	49	33
UBS com agentes contratantes privados para RH *	20	6	19	25	21	20
Oferta de ações em APS						
UBS que ofertam consultas médicas e consultas de enfermagem	99	94	99	99	99	99
% de UBS que ofertam consultas odontológicas	89	79	92	86	88	89
UBS realizam vacinação	85	81	93	80	76	80
UBS que dispensam medicamentos	70	79	83	54	71	56
Orientação comunitária e participação social						
UBS com definição de território de abrangência	85	77	84	89	89	83
UBS que realizam diagnóstico comunitário	66	59	68	68	67	62
UBS que realizam diagnóstico comunitário com periodicidade anual	49	56	51	48	41	43
UBS com Conselho Local de Saúde ativo	36	37	30	42	40	39
Coordenação e integração à rede regional						
Uso de prontuário eletrônico na UBS	87	65	84	92	98	95
Prontuário eletrônico compartilhado com outras UBS	70	42	64	76	85	83
Prontuário eletrônico compartilhado com serviços especializados públicos	19	6	8	29	36	20
Comunicação frequente das equipes com especialistas	41	42	37	46	43	41
A UBS recebe o resumo de alta hospitalar sempre ou quase sempre	28	20	21	42	27	19
UBS agenda a consulta/exame especializado no sistema de regulação e posteriormente informa a data ao usuário	59	52	46	73	71	53
Na UBS há registro/lista dos usuários encaminhados para outros pontos da rede	57	52	51	68	58	46
UBS monitora tempos de espera dos usuários encaminhados	34	31	30	43	36	25
UBS conta com serviços de referência para síndrome coronariana aguda	57	44	49	66	71	58
UBS conta com serviços de referência para acidente vascular encefálico	61	50	54	68	72	62

eSB: equipes de saúde bucal; eMulti: equipes multidisciplinares; eSF: equipes de saúde da família; PMM: Programa Mais Médicos; RH: recursos humanos.

Fonte: elaboração própria a partir de dados do Censo das UBS 2024 ^43,44^.

Nota: % em frequência relativa.

* Agentes contratantes privados: Organizações Sociais de Saúde (OSS), Organização da Sociedade Civil de Interesse Público (OSCIP), entidades filantrópicas, Organização Não-governamental (ONG), empresas privadas e cooperativas.

## Impactos da expansão da cobertura da ESF no acesso e na saúde da população

A expansão da ESF promoveu melhorias consistentes no acesso, na disponibilidade de fonte usual de cuidado e na situação de saúde da população, com redução de desigualdades sociais e regionais, conforme evidenciado por ampla literatura ^28^ ^,^ ^29^ ^,^ ^30^ ^,^ ^31^ ^,^ ^32^ ^,^ ^33^ ^,^ ^34^.

Resultados da *Pesquisa Nacional de Saúde* (PNS) 2019 confirmam o caráter equitativo da ESF: a cobertura foi mais elevada entre populações vulneráveis, sobretudo em domicílios de menor renda e escolaridade, alcançando 74% no primeiro quintil de renda contra 38% no quintil mais alto. A presença de fonte usual de cuidado, indicador indireto de acesso, foi maior entre cadastrados na ESF (79%) em comparação aos não cadastrados (73%), sendo a UBS o serviço de procura regular para 56% desses usuários ^4^.

Estudos demonstram impactos significativos da ESF sobre a morbimortalidade, sobretudo em contextos de cobertura elevada e estável: redução da mortalidade infantil ^35^ ^,^ ^36^, em menores de cinco anos e em condições sensíveis à APS em adultos e idosos ^37^. Os efeitos foram mais expressivos em grupos vulneráveis, contribuindo para a redução de desigualdades regionais e sociais em saúde ^29^ ^,^ ^38^.

Há também evidências de efeito sinérgico entre a ESF e o Programa Bolsa Família, com impacto na redução da pobreza, ampliação do acesso e queda da mortalidade infantil e por tuberculose ^32^ ^,^ ^39^ ^,^ ^40^. A ESF mostrou-se igualmente associada à queda de internações por condições sensíveis à APS em séries temporais abrangendo todos os municípios brasileiros ^31^ ^,^ ^34^.

A produção de evidências decorre majoritariamente de estudos ecológicos longitudinais, baseados em informações do Sistema de Informações sobre Mortalidade (SIM) e Sistemas de Informações Hospitalares (SIH). Mais recentemente, estudos de coorte com dados individualizados do Cadastro Único vinculados a bancos de dados em saúde reforçam a plausibilidade causal dos efeitos positivos da ESF sobre utilização de serviços e indicadores de saúde ^36^.

## Retrocessos

A trajetória da APS no Brasil acompanhou a institucionalização do SUS, marcada por avanços incrementais e retrocessos. Em um contexto de ruptura institucional, a revisão da PNAB em 2017 representou uma inflexão significativa ao extinguir a prioridade da ESF, até então eixo estruturante da APS ^5^.

Entre 2016 e 2022, políticas federais orientadas por governos de direita e extrema-direita promoveram retrocessos expressivos: fragilização da multiprofissionalidade com redução de ACS, extinção do financiamento aos NASF em 2019 e criação das chamadas equipes de atenção primária (eAP), compostas apenas por médico e enfermeiro com contratos por 20 ou 30 horas, desvinculadas da lógica territorial e comunitária da ESF, mas com financiamento equivalente. Essas medidas implicaram desmonte de quase três décadas de consolidação da ESF.

Entre 2019 e 2023, com o Previne Brasil, a centralidade no cadastramento individual como critério de financiamento substituiu a lógica de adscrição territorial e populacional, limitando ações coletivas, restringindo o acesso aos cadastrados e comprometendo os princípios de universalidade, integralidade e equidade. Tal arranjo fortaleceu uma concepção focalizada e seletiva de APS, em sintonia com uma cidadania restrita de caráter neoliberal, um “SUS mínimo” destinado aos pobres ^5^, deslocando a prioridade para equipes médico-centradas (eAP), comprometendo a integralidade do cuidado e interrompendo a trajetória de consolidação da ESF ^5^ ^,^ ^41^.

A retirada dos médicos cubanos do PMMB ao final de 2018 evidenciou a vulnerabilidade do modelo de provimento emergencial, gerando lacunas graves sobretudo em municípios rurais e remotos ^42^. Em 2019, o Programa Médicos pelo Brasil (PMpB) foi lançado sob gestão da Agência para o Desenvolvimento da Atenção Primária à Saúde (ADAPS), uma organização privada na forma de serviço social autônomo, suscitando críticas pela privatização da governança da APS e pela baixa efetividade de sua implementação inicial ^41^. O PMpB, no entanto, lançou seu primeiro edital somente em 2022 com abrangência restrita e o PMMB teve editais renovados no contexto da grave crise sanitária de COVID-19.

## Panorama atual da ESF

Ao longo de três décadas, o incentivo financeiro federal contínuo impulsionou a difusão da ESF em todo o país. Em 2024, com 54,6 mil equipes de ESF, 300 mil ACS e 32 mil eSB, estima-se uma cobertura potencial da ESF de 78% da população brasileira, embora os dados oficiais reportem 100% de cobertura da APS (Tabela 1).

A expansão, contudo, apresenta forte heterogeneidade territorial, especialmente entre áreas urbanas e rurais. Embora, em 88% das UBS do país atuem eSF, 67% dispõem de apenas uma eSF, 11%, de duas equipes e 10%, de três ou mais equipes. As UBS com apenas uma equipe predominam em municípios pequenos, em contraste, municípios maiores concentram UBS com duas ou mais equipes ^43^ (Tabela 2). Estabelecimentos com maior número de equipes em geral contemplam infraestrutura com maior diversidade de equipamentos, serviços de apoio e outros profissionais com maior capacidade resolutiva. Esta heterogeneidade afeta a equidade. O contexto brasileiro com cerca de 70% dos municípios com menos de 20 mil habitantes exige arranjos diferenciados para promover maior capacidade resolutiva na APS, em localidades menores ainda que com perda de economia de escala.

As UBS ofertam ações abrangentes ao longo do curso de vida (saúde da criança, saúde materna, saúde da mulher, saúde do idoso), incluindo promoção, prevenção e cuidado, respostas a demandas agudas e cuidado a portadores de agravos crônicos (hipertensão, diabetes, tuberculose) entre outros. Porém, o escopo é desigual entre territórios, sendo mais amplo em centros urbanos, embora médicos rurais frequentemente realizem maior número de procedimentos ^44^ ^,^ ^45^ ^,^ ^46^.

A territorialização e a orientação comunitária, bases da ESF, ainda carecem de consolidação: 85% das UBS possuem território de referência, mas apenas metade realiza diagnóstico comunitário anual e pouco mais de um terço mantém conselhos locais ativos ^44^ (Tabela 2). A atuação comunitária e o diagnóstico comunitário participativo anual viabilizam o reconhecimento do território, de seus recursos potenciais, carências de serviços e dificuldades de acesso às ações de promoção da saúde. Permitem, assim, a programação das ações mais efetivas às necessidades populacionais, a partir de informações atualizadas do contexto local histórico, cultural, social, ambiental e sanitário, conforme riscos e vulnerabilidades.

No entanto, a ação comunitária revela desigualdades regionais e fragilidades, tendo maior alcance na Região Nordeste, como evidenciado no enfrentamento da pandemia de COVID-19 ^47^. Esse processo está intimamente vinculado à ação dos ACS no contexto domiciliar e comunitário, que em muitos lugares tem sofrido redução devido à sobrecarga de atividades administrativas nas UBS ^48^.

As UBS são formalmente o primeiro ponto de contato e a principal porta de entrada ao sistema de saúde e pretende-se que cumpram a função de filtro (*gatekeeper*) para atenção especializada coordenando o cuidado, no entanto, a integração com a rede especializada e hospitalar enfrenta barreiras estruturais, especialmente nas regiões Norte e Nordeste, onde a escassez de serviços públicos e a concentração de recursos em áreas urbanas reforçam desigualdades históricas.

A partir de 2023, sob o governo Lula e a liderança da ministra da Saúde Nísia Trindade, iniciou-se processo de reconstrução do SUS e valorização da ESF como modelo prioritário da APS. Foram retomados o financiamento multiprofissional, agora com as eMulti, a expansão e reformulação do PMMB, com maior ênfase na formação de especialistas em APS e fixação profissional com permanência de até oito anos com novos incentivos financeiros. Em 2025, o PMMB conta com mais de 25 mil médicos atuando em 43% das UBS do país, presentes em 80% dos municípios, configurando-se como pilar estratégico para o fortalecimento da APS e para a redução das desigualdades em saúde. Além disso, o credenciamento de novas eSB, interrompido nos anos anteriores, que distingue a APS brasileira no contexto global, foi retomado.

As eMulti, estão, agora, vinculadas a diferentes modalidades de equipes de APS, com composição ampliada, incluindo novas especialidades médicas e recursos de telessaúde. Apesar do novo arranjo e da autonomia municipal para definir sua composição, a cobertura das eMulti permanece limitada, com desafios para consolidar práticas interprofissionais efetivas e garantir apoio sistemático às UBS.

## Desafios permanentes

Apesar da retomada da prioridade do modelo da ESF a partir de 2023, persistem problemas estruturais não equacionados pelas reformas incrementais. Entres estes desafios, destacam-se a seguir aqueles relacionados ao financiamento adequado para a universalização da cobertura de ESF com qualidade; à disputa dos recursos públicos e os riscos da mercantilização na APS; à força de trabalho com formação adequada e inserção com estabilidade na APS; ao alcance da equidade em saúde em especial para o enfrentamento do racismo estrutural; e à coordenação do cuidado e integração à rede para a garantia do cuidado integral.

### Financiamento: recursos suficientes para universalizar a ESF com qualidade 

A universalização da ESF com qualidade exige recursos suficientes para garantir UBS bem estruturadas, equipes multiprofissionais competentes e adequadas à magnitude da população de referência e elevada capacidade resolutiva. O financiamento da APS, de responsabilidade tripartite, tem recaído majoritariamente sobre os municípios, em razão da estagnação da União sob políticas de austeridade e da baixa participação das Unidades da Federação, acentuando conflitos intergovernamentais.

O novo modelo de cofinanciamento federal, vigente a partir de 2024, que poderia ser denominado “Mais Saúde da Família”, restabelece a prioridade para a ESF, com valores fixos mais expressivos por equipe e incentivos associados à vulnerabilidade social da população adscrita, incluindo seu dimensionamento por equipe. Além disso, incorpora indicadores de qualidade pactuados em negociação tripartite, reafirmando a necessidade de alinhar financiamento e modelo assistencial baseado em territorialização, multiprofissionalidade e integração em rede. Contudo, a coexistência com mais de 6 mil equipes de eAP (Tabela 1) e programas para acesso a especialidades, necessários, mas ainda sem integração bem definida, tensionam o sistema e evidenciam disputas entre modelos assistenciais.

Além disso, o subfinanciamento crônico do SUS, com gastos públicos em saúde inferiores a 4% do produto interno bruto (PIB) - muito abaixo dos cerca de 7% investidos em países com sistemas universais, permanece um obstáculo central à expansão e qualificação da APS. Conforme argumenta o relatório da Comissão Global de Saúde da Lancet sobre o financiamento da APS, os países precisam investir mais e investir melhor na APS ^49^. Porém, nossa perspectiva é muito diferente daquela concepção focalizada e seletiva preconizada no documento para os países de renda baixa e média, que deveriam se ater a uma cobertura universal apenas de serviços mínimos em APS, que condicionaria a ampliação de outros serviços ^49^.

Para alcançar a universalização da ESF, no marco dos princípios do SUS de universalidade, integralidade e equidade, se requer revisão do arcabouço fiscal e a proteção de recursos constitucionais suficientes para o SUS, garantindo transferências federais em APS para os municípios brasileiros adequadas às necessidades locais ^50^. Ademais do custeio, investimentos são necessários para viabilizar a melhoria da infraestrutura, de equipamentos clínicos, saúde digital e telessaúde, a valorização e a educação permanente dos profissionais, de modo a ampliar a capacidade resolutiva da APS, reduzir encaminhamentos desnecessários e efetivar a coordenação do cuidado com serviços especializados.

### Mercantilização na APS

A APS, historicamente menos permeada por processos de mercantilização dentro do SUS, vem apresentando crescente inserção de agentes privados na provisão de serviços ^51^. Embora a maioria das UBS permaneça sob propriedade municipal (85%), observa-se expansão da gestão por Organizações Sociais de Saúde (OSS), Organização da Sociedade Civil de Interesse Público (OSCIP) e entidades filantrópicas, especialmente em grandes centros urbanos. O Censo das UBS de 2024 indica que 20% das unidades já operam sob arranjos de gestão privada, tendência que sinaliza a ampliação da participação do setor privado no nível primário de atenção (Tabela 2).

Adicionalmente, a criação da Agência Brasileira de Apoio à Gestão do SUS (AgSUS), um serviço social autônomo, reformulando a ADAPS, criado para dar suporte operacional às políticas do Ministério da Saúde, especialmente nas áreas de Saúde Indígena e APS, introduz outras possibilidades de contratação privada em escala federal, reforçando as incertezas quanto à manutenção da APS como bem público.

Esses movimentos de terceirizações, parcerias público-privadas ainda que em parte sejam organizações instituídas como sem fins de lucro, parecem inserir os serviços de APS em um cenário mais amplo de mercantilização da saúde, com transferência de recursos públicos para entes privados, com potenciais impactos sobre a natureza pública do SUS ^52^.

### Força de trabalho para a ESF: formação, vínculos e carreira

A consolidação da ESF depende da disponibilidade de profissionais qualificados, com vínculos estáveis e permanência nas equipes, condição essencial para garantir a longitudinalidade do cuidado. Desde os anos 1990, políticas de formação e educação permanente em saúde buscaram fortalecer a atuação na APS, mas enfrentaram descontinuidades, frágil integração entre educação, serviço e comunidade e elevada rotatividade profissional ^53^.

A formação de médicos generalistas historicamente foi limitada nas escolas médicas, com tardio reconhecimento da Medicina de Família e Comunidade como especialidade em 2002 e insuficiente valorização profissional, refletida em vínculos precários e remuneração desigual.

De todo modo, ao longo do tempo observou-se ampliação da formação multiprofissional para APS com especializações, residências de Enfermagem de Família e Comunidade, de Medicina de Família e Comunidade, e multiprofissionais, e um programas de mestrado profissional em rede nacional, a exemplo do ProfSaúde, financiado pelo Ministério da Saúde e algumas turmas pelo Ministério da Educação, dirigido inicialmente apenas para médicos, passou a incluir outras profissões.

As iniciativas de formação multiprofissional visam promover a interprofissionalidade. A atuação colaborativa interprofissional, ao integrar decisões e responsabilidades, constitui estratégia central para enfrentar a complexidade da multimorbidade e do cuidado integral, com destaque para a ampliação do escopo da enfermagem e práticas avançadas centradas na pessoa ^54^. Contudo, a institucionalização dessas práticas enfrenta barreiras estruturais, como a hegemonia médico-centrada e hierarquias de gênero, raça e classe ^55^.

A fixação profissional permanece um desafio crítico para a qualificação da APS. Em 2024, apenas 33% das UBS contavam com pelo menos um médico servidor público, e 42% com médicos vinculados ao PMM, em contratos temporários sem garantias de carreira. A persistência de vínculos precários limita a continuidade do cuidado e fragiliza o modelo assistencial. A ausência de uma carreira nacional para a APS e o SUS reflete constrangimentos fiscais, reformas trabalhistas e previdenciárias e a Lei de Responsabilidade Fiscal, que restringem contratações estáveis ^56^.

Assim, o fortalecimento da ESF requer políticas consistentes de formação e educação permanente em APS, para proporcionar vínculos de trabalho estáveis com implementação de carreiras públicas e maior tempo de atuação dos profissionais nas equipes, para a valorização do trabalho interprofissional, articuladas a um projeto nacional de fortalecimento do SUS.

### Equidade em saúde e enfrentamento do racismo estrutural na APS

O racismo estrutural constitui um determinante central das desigualdades em saúde no Brasil, afetando de forma desproporcional a população negra e parda, com marcante interseccionalidade com gênero e classe social ^57^. Evidências apontam que a discriminação nos serviços de saúde gera barreiras econômicas, organizacionais e culturais, restringindo o acesso oportuno e qualificado ^58^.

A instituição da Política Nacional de Saúde Integral da População Negra (PNSIPN), publicada em 2007 e revisada em 2017, representou um marco no reconhecimento das desigualdades étnico-raciais e do racismo institucional como determinantes sociais da saúde ^59^ ^,^ ^60^. No entanto, sua implementação permanece incipiente: em 2024, apenas 55% das UBS declararam adotar estratégias contra o racismo, e somente 13% realizaram formações específicas sobre a política no último ano ^43^.

A ESF, por sua abordagem territorial e comunitária, apresenta potencial privilegiado para promover atenção integral equitativa, fortalecer vínculos com políticas sociais e incorporar práticas intersetoriais voltadas ao enfrentamento das desigualdades raciais. Contudo, sua efetividade depende da formação continuada dos profissionais, da institucionalização da PNSIPN e da transversalidade das ações de saúde com políticas de proteção social, de equidade de gênero, classe e raça/etnia ^58^.

A consolidação de políticas customizadas e interseccionais é imprescindível para reduzir desigualdades e afirmar o SUS como sistema universal, integral e promotor de justiça social ^58^.

### Coordenação, integração à rede e continuidade do cuidado na APS

A qualificação da APS requer capacidade efetiva de coordenação do cuidado, integração às redes regionais do SUS e garantia da continuidade da atenção. A Política Nacional de Atenção Especializada em Saúde (PNAES), de 2023, reafirma a APS como porta de entrada preferencial, centro de comunicação da Rede de Atenção à Saúde (RAS) e principal responsável pela ordenação do acesso, com fortalecimento de sua capacidade clínica e corresponsabilização pelo cuidado ^61^.

A literatura destaca a centralidade da APS em sistemas de saúde de alto desempenho ^49^. Contudo, no Brasil, persistem barreiras à sua função de coordenadora, relacionadas à fragmentação assistencial, escassez de serviços especializados e falhas na articulação interprofissional e informacional.

A continuidade do cuidado com qualidade envolve a continuidade informacional que inicia na UBS com o prontuário eletrônico, tendo ocorrido importante ampliação do uso de prontuários eletrônicos na APS na última década. Em 2012, estava disponível em apenas 14% das UBS, sendo mencionado por 39% dos serviços em 2018 no terceiro ciclo do PMAQ-AB ^19^, alcançando 89% das UBS em 2024. O compartilhamento do prontuário Eletrônico do Cidadão com outras UBS é frequente (70%), no entanto, somente menos de um quinto das UBS compartilhavam registros com serviços especializados públicos (18%) em 2024. A ausência de interoperabilidade e a baixa frequência de recebimento de resumos de alta hospitalar (menos de um terço das UBS) comprometem a continuidade informacional. Além disso, a comunicação entre equipes da ESF e especialistas é frequente em apenas 41% das UBS (Tabela 2).

Os mecanismos de regulação também revelam fragilidades: somente 57% das UBS registravam encaminhamentos e 34% monitoravam tempos de espera. Em 44% das UBS, o usuário recebe ficha física para buscar atendimento por conta própria, evidenciando baixa responsabilização da APS pelo cuidado especializado (Tabela 2).

Apesar da abrangência superior a 85% para serviços de referência em câncer de mama e colo do útero, gestação de alto risco, parto e saúde mental, persistem lacunas críticas na definição de serviços de referência para doenças cardiovasculares e em regiões como Norte e Nordeste ^43^.

Consolidar a APS como coordenadora da RAS exige a ampliação do escopo clínico e da capacidade resolutiva das equipes ESF; integração operacional das UBS com centrais de regulação para reduzir tempos de espera e percursos ineficazes; interoperabilidade plena dos prontuários eletrônicos e com os demais sistemas de informação; comunicação interprofissional contínua; definição clara de fluxos regionais e serviços de referência, com expansão da oferta especializada pública e fortalecimento da responsabilização da APS pelos encaminhamentos e continuidade do cuidado ^62^.

## Considerações finais

Os avanços na cobertura populacional não superaram vazios assistenciais e lacunas na plena implementação do modelo assistencial da ESF de orientação comunitária e territorializada e integrada à rede. Esta abordagem abrangente da ESF sustenta-se em equipes multiprofissionais com trabalho interprofissional e colaborativo; enfoque territorial e comunitário; vigilância em saúde e responsabilização da saúde da população de determinado território; participação social e mediação de intersetorialidade; escopo ampliado das ações individuais e coletivas; coordenação do cuidado e integração à rede regionalizada; abordagem intercultural; e valorização da força de trabalho na APS. 

Urge preencher vazios assistenciais, incorporar tecnologia para aumento da capacidade resolutiva da APS com qualidade, aprimorar a organização e integração da rede, garantir financiamento adequado e sustentável com critérios que promovam a equidade e qualidade, e que respondam às necessidades de saúde da população ^62^. Somente a articulação sistêmica entre APS, serviços especializados e hospitalares, em redes regionalizadas e equitativas, permitirá superar a fragmentação atual e consolidar o SUS como sistema universal, integral e resolutivo ^62^.

A plena implementação do modelo assistencial da ESF requer retomar e fortalecer os seus princípios constitutivos de participação social, abordagem territorial e comunitária, integração da vigilância em saúde com cuidado individual oportuno, promoção, prevenção e cuidado, integração à rede, e mediação de ações intersetoriais para promoção da saúde e redução de desigualdades de gênero, étnico-raciais e sociais.

Sem dúvidas, a consolidação da ESF exige superar a vulnerabilidade institucional a mudanças políticas e reafirmar os princípios constitucionais do SUS como diretriz inegociável da política de APS.

## Data Availability

As fontes de informação utilizadas no estudo estão indicadas no corpo do artigo.
